# APP family in inhibitory neurons controls inhibitory recruitment and short-term plasticity in the hippocampus

**DOI:** 10.1186/s13041-026-01296-0

**Published:** 2026-03-29

**Authors:** Sang Hun Lee, Jongkyun Kang, Chen Zhang, Vadim Y. Bolshakov, Jie Shen

**Affiliations:** 1 Department of Neurology, Brigham & Women’s Hospital, Harvard Medical School, Boston, MA 02115 USA; 2 Department of Psychiatry, McLean Hospital, Harvard Medical School, Belmont, MA 02478 USA; 3 Program in Neuroscience, Harvard Medical School, Boston, MA 02115 USA

**Keywords:** Alzheimer’s disease, Amyloid Precursor Protein, Inhibitory neuron, Conditional triple knockout, Hippocampus, Schaffer collateral, Short-term synaptic plasticity, Long-term potentiation

## Abstract

Amyloid precursor protein (APP) is associated with both familial and sporadic forms of Alzheimer’s disease. We previously reported that APP and its family members, amyloid precursor-like proteins 1 and 2 (APLP1 and APLP2), regulate intrinsic neuronal excitability and synaptic plasticity in excitatory principal neurons, though APP family is dispensable for neuronal survival. However, the physiological role of APP family in inhibitory interneurons remains poorly understood. Here, we use our previously characterized floxed *APP*, *APLP1*, *APLP2* and *GAD2-Cre* alleles to generate inhibitory neuron-specific conditional triple knockout (IN-*APP/APLP1/APLP2* cTKO) mice. Our electrophysiological analysis of acute hippocampal slices revealed that IN-*APP/APLP1/APLP2* cTKO CA1 pyramidal neurons exhibit increased amplitudes of evoked GABA_A_ receptor-mediated inhibitory postsynaptic currents (IPSCs), while basal spontaneous IPSC frequency and amplitude remain unchanged. At Schaffer collateral (SC)–CA1 synapses, short-train frequency facilitation is enhanced in slices from IN-*APP/APLP1/APLP2* cTKO mice, whereas paired-pulse facilitation (PPF) and long-term potentiation (LTP) are normal. Consistent with a cell-autonomous interneuron defect, basal excitatory transmission, measured by spontaneous and miniature excitatory postsynaptic currents (EPSCs) in CA1 pyramidal neurons, is unaltered. These data show that APP family in inhibitory interneurons regulates activity-dependent inhibitory output without overtly perturbing baseline glutamatergic transmission and thus, indirectly shapes short-term facilitation at SC–CA1 synapses. Together with our earlier findings in excitatory neuron-specific *APP/APLP1/APLP2* cTKO mice showing intrinsic hyperexcitability, enhanced short-term facilitation, and impaired LTP, these results suggest that the APP family modulates inhibitory and excitatory functions at SC–CA1 synapses through complementary mechanisms in principal neurons and interneurons.

## Introduction

Balanced excitation and inhibition is fundamental for the control of information flow and synaptic plasticity in hippocampal circuits [1, 2]. Mounting evidence from both human and animal studies shows that when inhibitory interneuron function weakens, neural networks become hypersynchronous, gamma-band rhythms degrade, and cognition suffers, underscoring the need for defined interneuron-intrinsic programs stabilizing circuit dynamics [3–5]. Studies across scales, including single cells, ensembles, and whole networks, indicate that hyperexcitability and subclinical epileptiform activity can emerge early in Alzheimer’s disease (AD) and are closely linked to cognitive decline [4, 6–9]. Interventions that restore inhibitory function or otherwise temper aberrant activity have been shown to normalize oscillatory patterns and improve behavioral readouts in experimental models, supporting a causal role for inhibitory control in disease-relevant circuit dysfunction [3, 5, 10, 11]. In this context, cell-type–specific genetics have begun to define interneuron regulators of hippocampal physiology. Interneuron-specific *Presenilin* conditional double-knockout (IN-*PS* cDKO) mice show decreased GABAergic efficacy and enhanced Schaffer collateral (SC)–CA1 plasticity, and later develop age-dependent interneuronal loss and gliosis, demonstrating a requirement for Presenilin (PS) within inhibitory neurons to maintain inhibitory output and circuit balance [12, 13].

In parallel, the amyloid precursor protein (APP) family (APP, APLP1, APLP2) has emerged as a key organizer of synaptic function. In excitatory neurons, forebrain-restricted triple deletion impairs hippocampal long-term potentiation (LTP) and learning and increases intrinsic excitability, with effects linked to altered Kv7 channel control, without causing neurodegeneration; together, these findings establish APP family as physiological modulators of excitability and plasticity rather than survival factors [14]. Moreover, APP-derived soluble ectodomains (sAPPα) enhance synaptic plasticity through activity-dependent protein synthesis and AMPA receptor trafficking [15, 16]. Collectively, this work positions the APP family as a synapse-centric signaling system with the capacity to tune neurotransmitter release dynamics, plasticity, and neuronal excitability.

Despite extensive studies in principal cells, it remains unclear whether APP family signaling acts cell-autonomously in interneurons to determine inhibitory strength and short-term dynamics within intact neural circuits. Several lines of evidence motivate this question. First, network-level phenotypes in AD models are highly sensitive to inhibitory tone and interneuron health, suggesting that interneuron-intrinsic modulators can exert outsized circuit-level effects [4]. Second, APP family include validated synaptic adhesion proteins (notably APLP1) that could influence presynaptic coupling and recruitment at inhibitory synapses [17]. Third, prior interneuron-specific γ-secretase disruption (IN-*PS* cDKO) decreased inhibitory drive and increased plasticity [12], implying that mechanistically distinct, parallel APP family pathways might shape inhibition independently of γ-secretase.

Here, we address this knowledge gap by selectively inactivating *APP*, *APLP1*, and *APLP2* in GABAergic interneurons (IN-*APP/APLP1/APLP2* cTKO) using previously characterized floxed *APP/APLP1/APLP2* alleles and *GAD2-Cre* followed by assaying hippocampal synaptic function. We focused on CA1 circuitry to enable direct comparison with our excitatory-neuron cTKO studies [14]. Our results suggest a cell-type–specific contribution of the APP family in inhibitory neurons: increased recruitment of evoked GABAergic inhibition and heightened short-term frequency facilitation at SC–CA1 synapses, without detectable changes in paired-pulse facilitation (PPF), LTP, or basal excitatory transmission in cTKO mice. Taken together, these findings suggest that the APP family plays an important role in interneurons by shaping inhibitory output and synaptic facilitation in the hippocampus, thereby extending APP function beyond principal cells.

## Methods

### Generation of inhibitory neuron-specific *APP/APLP1/APLP2* cTKO mice

All mice were housed in humidity- and temperature-controlled rooms maintained on a 12:12 h light: dark cycle and were given standard rodent chow and water. To generate inhibitory neuron-specific *APP/APLP1/APLP2* conditional triple knockout (IN-cTKO) mice, we crossed floxed *APP*, *APLP1*, and *APLP2* alleles, which were thoroughly characterized previously and were used to generate excitatory neuron-specific *APP/APLP1/APLP2* cTKO mice [14], with *GAD2-IRES-Cre* knockin (KI) mice (The Jackson Laboratory, 010802, RRID: IMSR_JAX:010802), which we previously characterized to generate interneuron-specific *PS* cDKO mice [18], to obtain *fAPP/+; fAPLP1*/+; *fAPLP2/+; GAD2-IRES-Cre/+* mice, which were then bred with *fAPP/fAPP; fAPLP1/fAPLP1; fAPLP2/fAPLP2* mice to generate *fAPP/fAPP; fAPLP1/fAPLP1; fAPLP2/fAPLP2; GAD2-IRES-Cre/+* (IN-*APP* cTKO) mice. IN-*APP* cTKO and littermate control (*fAPP/fAPP; fAPLP1/fAPLP1; fAPLP2/fAPLP2*) mice used in the study were obtained from breeding *fAPP/fAPP; fAPLP1/fAPLP1; fAPLP2/fAPLP2* mice together with *fAPP/fAPP; fAPLP1/fAPLP1; fAPLP2/fAPLP2; GAD2-IRES-Cre/+* mice. Both IN-*APP/APLP1/APLP2* cTKO male and female mice are viable and fertile with normal size litters. Genotyping was performed at postnatal days 10–12, and the progeny of intercrosses of IN-*APP* cTKO and control mice were found at the expected Mendelian ratio.

Both male and female mice were used in each experiment. IN-*APP/APLP1/APLP2* cTKO and littermate control mice were maintained in the C57BL/6 J 129 hybrid genetic background. All procedures were approved by the IACUC committees of Harvard Medical School and Brigham and Women’s Hospital, and conform to the USDA Animal Welfare Act, PHS Policy on Humane Care and Use of Laboratory Animals, the “ILAR Guide for the Care and Use of Laboratory Animals” and other applicable laws and regulations.

### PCR genotyping

Tail genomic DNA was extracted at postnatal days 10–12, and genomic PCR was performed to determine the presence of the deleted, the floxed, and/or wild-type allele [14]. For *APP*, the following primers were used: 5’-GGCCTTCTAGGTTGCTTTCTATTGC (RB1101, forward primer at ~ 1500nt upstream of exon 1), 5’-AAGCAGTTTCTGCCACTGCCCAGTT (RB1102, reverse primer at ~ 1300nt upstream of exon 1), and 50-AAGAGTCCTGGACGTCCAGGTTGA (RB1105, reverse primer at ~ 800nt downstream of exon 1). The PCR products from RB1101 and RB1102 are 180 bp and 222 bp, which represent the wild-type and the floxed *APP* alleles, respectively, whereas the PCR products from RB1101 and RB1105 is 377 bp, which represents the deleted *APP* allele.

For *APLP1*, the following primers were used: 5’-GCCACATGAGTCATGGACCTTGAAT (RB1110, forward primer at ~ 1500nt upstream of exon 1), 5’-AGGACTTAGGACATCATCGCTACTG (RB1111, reverse primer at ~ 1300nt upstream of exon 1), and 5’-TCTCATTGGGCTCCATCACTTACTG (RB1112, reverse primer at ~ 400nt downstream of exon 2). The PCR products from RB1110 and RB1111 are 167 bp and 203 bp, which represent the wild-type and the floxed *APLP1* alleles, respectively, whereas the PCR product from RB1110 and RB1112 is 461 bp, which represents the deleted *APLP1* allele.

For *APLP2*, the following primers were used: 5’-ATTCTAGGGCCTCTGGATTGA (AH08166, forward primer at ~ 1500nt upstream of exon 1), 5’-TAGTGGGCAGAGTGGGACAGTAAG (AH08164, reverse primer at ~ 1100nt upstream of exon 1), and 5’-GGAGACGCAGATCGGGAGCT (AH09209, reverse primer at ~ 40nt downstream of exon 1). The PCR products from AH08166 and AH08164 are 385 bp and 425 bp, which represent the wild-type and the floxed *APLP2* alleles, respectively, whereas the PCR from AH08166 and AH09209 is 188 bp, which represents the deleted *APLP2* allele.

The primers used in the PCR to differentiate the wild-type or the *GAD2-IRES-Cre* KI allele are JKM028 (5’-CTTCTTCCGCATGGTCATCT, forward primer in the coding sequences of *GAD2* exon 16), JKM029 (5’-CACCCCACTGGTTTTGATTT, reverse primer in the 3’ UTR of *GAD2* exon 16), and JKM030 (5’-AAAGCAATAGCATCACAAATTTCA, reverse primer in the SV40 polyA signal sequences of *IRES-Cre*, inserted immediately following the stop codon of the *GAD2* gene in exon 16). The 250 bp PCR product (from JKM028-JKM029) represents the wild-type *GAD2* allele, whereas the 352 bp fragment (from JKM028-JKM030) represents the C*re* KI allele.

### Western analysis

The neocortex, hippocampus, and striatum were dissected from the harvested brain. Fresh tissues were homogenized in an ice-cold stringent RIPA buffer [50 mM Tris-Cl (pH 7.6), 150 mM NaCl, 0.5 mM EDTA, 1% NP40, 0.5% sodium deoxycholate, 0.1% SDS, 1mM PMSF supplement with protease inhibitor cocktail and phosphatase inhibitor cocktail (Sigma)], followed by sonication. Homogenates were centrifuged at 14,000 rpm for 20 min at 4 °C to separate supernatants (RIPA buffer-soluble fraction). Equal amounts (10–40 µg per lane) of total proteins from each preparation were loaded and separated on NuPAGE gels (Invitrogen), and transferred to nitrocellulose membranes. The membranes were blocked in 5% skim milk/TBS for 1 h, and incubated at 4 °C overnight with specific primary antibodies. The primary antibodies used were rabbit anti-APP (Sigma-Aldrich, A8717, RRID: AB_258409), rabbit anti-APP-Y188 (Abcam, ab32136, RRID: AB_2289606), rabbit anti-APLP1 (CT11, gift of D. Walsh), rabbit anti-APLP2 (D2-II, gift of D. Walsh), or mouse anti-β-actin (#3700, Cell Signaling, 1/4000, RRID: AB_2242334). Membranes were then incubated with secondary antibodies, which was either goat anti-rabbit IRdye680 (926-68071, LI-COR Bioscience, 1/20000, RRID: AB_10956166), or goat anti-mouse IRdye800 (926-32210, LI-COR Bioscience, 1/20000, RRID: AB_621842). Signals were quantified using the Odyssey Infrared Imaging System (LI-COR Biosciences).

### Histological analysis

Mice were anesthetized with ketamine (100 mg/kg) + xylazine (10 mg/kg) + acepromazine (3 mg/kg), and transcardially perfused with phosphate-buffered saline solution (PBS, pH 7.4) containing 0.25 g/L heparin (Sigma) and 5 g/L procaine (Sigma). Brains were post-fixed in 4% formaldehyde in PBS (Electron Microscopy Sciences) at 4˚C overnight and then processed for paraffin embedding following standard procedures. Serial sagittal Sect. (10 μm) were obtained using Leica RM2235. Immunofluorescence staining and analysis were performed as previously described [19]. Briefly, paraffin-embedded sagittal brain sections were deparaffinized and rehydrated, then subjected to permeabilization with a solution containing 0.1% Triton X-100 and 0.1% sodium citrate in PBS. Antigen retrieval was performed by microwaving the sections for 10 min in 10 mM sodium citrate buffer (pH 6.0). Sections were then blocked with a solution containing 5% normal goat serum (Vector Laboratories) for 1 h. After blocking, sections were incubated with primary antibodies overnight at 4˚C. The primary antibodies used were mouse anti-GAD67 (MAB5406, MilliporeSigma, 1/500, RRID: AB_2278725) and rabbit anti-APP-Y188 (ab32136, Abcam, 1/250, RRID: AB_2289606). Sections were then incubated for 1 h with fluorophore conjugated secondary antibodies, Alexa Fluor 488 goat anti-rabbit IgG (A-11034, Thermo Fisher Scientific, 1/250, RRID: AB_2576217) and Alexa Fluor 555 goat anti-mouse IgG (A-21424, Thermo Fisher Scientific, 1/250, RRID: AB_141780) at room temperature. Fluorescence images were taken and analyzed by FV1000 confocal microscope system (Olympus). 

### Preparation of brain slices for electrophysiology

Hippocampal slices were prepared from both male and female IN-*APP/APLP1/APLP2* cTKO and littermate control mice at 3 months of age. Mice were decapitated after being anesthetized with ketamine (100 mg/kg) + xylazine (10 mg/kg) + acepromazine (3 mg/kg). The brain was removed and placed in ice-cold (4 °C) oxygenated (95% O_2_/5% CO_2_) high sucrose and magnesium solution containing (in mM) the following: 200 Sucrose, 25 NaHCO_3_, 10 Glucose, 3 KCl, 1.25 NaH_2_PO_4_, 1.2 Na-pyruvate and 0.4 Na-ascorbate, 7 MgCl_2_, and 0.5 CaCl_2_. Horizontal hippocampal slices (400 μm thick) were prepared using a vibratome (VT1200S, Leica, Germany), and transferred to an incubation chamber having oxygenated artificial cerebrospinal fluid (ACSF) containing (in mM) the following: 125 NaCl, 3 KCl, 1.25 NaH_2_PO_4_, 1 MgCl_2_, 2 CaCl_2_, 25 NaHCO_3_, 10 Glucose, 1.2 Na-pyruvate and 0.4 Na-ascorbate, adjusted to 310 ± 5 mOsm (pH 7.4). The slices were allowed to recover at 34 °C for 1 h and then placed in a recording chamber constantly perfused with heated ACSF (30 ± 1 °C) and gassed continuously with 95% O_2_ and 5% CO_2_. The flow rates of bathing solution and the volume of the recording chamber for slices were 2.2 ml/min and 1.2 ml, respectively. Hippocampal slices were visualized using an upright microscope equipped with differential interference contrast (DIC) optics (BX51WI, Olympus, Japan). The DIC optics was used for visualization of neurons in the course of whole-cell recordings. In a subset of experiments, the following drugs were used at the following concentrations via bath application or adding intracellular recording solutions: Picrotoxin (100 µM, Tocris #1128), Bicuculline methochloride (20 µM, Tocris #0131), D-AP5 (50 µM, Tocris #0106), NBQX disodium salt (10 µM, Tocris #1044), QX314 chloride (5 mM, Tocris #2313) and Tetrodotoxin (TTX: 1 µM, Tocris #1069).

### Electrophysiological analysis

In whole-cell patch-clamp experiments, evoked inhibitory postsynaptic currents (IPSCs) for input-output relations were elicited by a unipolar stimulation electrode positioned near the recorded CA1 pyramidal neuron in stratum pyramidale (approximately 100 μm from the soma) and recorded at a holding potential of − 70 mV in the presence of blockers of AMPA (10 µM NBQX) and NMDA (50 µM D-AP5) receptors. The stimulation pulses ranging from 30 to 110 µA were delivered using a stimulus isolation unit (A365, World Precision Instruments, USA) with a unipolar metal microelectrode. Spontaneous inhibitory postsynaptic currents (sIPSCs) were recorded from CA1 pyramidal neurons in voltage-clamp mode at a holding potential of − 70 mV in the presence of blockers of AMPA (10 µM NBQX) and NMDA (50 µM D-AP5) receptors. The recording pipettes (3–5 MΩ) were filled with a solution containing (in mM) the following: 130 KCl, 10 phosphocreatine, 20 HEPES, 4 MgATP, 0.3 NaGTP, 5 QX314 and 0.2 EGTA with the pH adjusted to 7.30 with KOH (295–300 mOsm). Spontaneous or miniature excitatory postsynaptic currents (sEPSCs or mEPSCs) were recorded from CA1 pyramidal neurons in voltage-clamp mode at a holding potential of − 70 mV in the presence of 100 µM picrotoxin for the blockade of GABA_A_ receptor without or with 1 µM TTX, respectively. The recording pipettes (3–5 MΩ) were filled with a solution containing (in mM) the following: 120 K-gluconate, 10 KCl, 20 HEPES, 4 MgATP, 0.3 NaGTP, 10 phosphocreatine, and 0.2 EGTA with the pH adjusted to 7.30 with KOH (295–300 mOsm). The series resistance (Rs) after establishing whole-cell configuration was between 15 and 25 MΩ. IPSC or EPSC recordings with > 20% series resistance changes were excluded from the data analysis.

For extracellular field recordings, stimulation pulses were delivered with a stimulus isolation unit (A365, World Precision Instruments, USA) using a unipolar metal stimulation microelectrode. Field excitatory postsynaptic potentials (fEPSPs) were recorded in current-clamp mode with ACSF-filled patch pipettes (1.5–2 MΩ). All fEPSPs were recorded with a stimulation strength that yielded ~ 50% of the maximal response. Maximal responses were determined from input-output curves by incrementally increasing stimulation until the fEPSP slope approached a plateau while avoiding population spike contamination. Data were collected with a MultiClamp 700B amplifier (Molecular Devices, USA) and digitized at 10 kHz using the A/D converter DIGIDATA 1322 A (Molecular Devices, USA). Data were acquired and analyzed using a custom program written with Igor Pro software (Version 6.3; Wave-Metrics) and Clampfit (Version 10.3; Molecular device). Paired-pulse facilitation (PPF) was measured as the ratio of the second fEPSP slope relative to the first fEPSP slope, evoked by two identical presynaptic stimuli. Synaptic facilitation was measured as the percentage of the fEPSP slope versus the first fEPSP slope at a given stimulus train in individual slices. In long-term potentiation (LTP) recordings, after baseline responses were collected every 15 s for 15 min, LTP was induced by five episodes of theta burst stimulation (TBS) delivered at 0.1 Hz. Each episode contained ten stimulus trains (5 pulses at 100 Hz) delivered at 5 Hz. To generate summary graphs (mean ± SEM), individual experiments were normalized to the baseline, and four consecutive responses were averaged to generate 1 min bins. These were then averaged together to generate the final summary graphs.

### Data quantification and statistical analysis

Data acquisition and quantification were performed in a genotype blind manner. All statistical analysis was performed using Prism (Version 10; GraphPad software), Excel (Microsoft), Igor Pro (Version 6.3; Wave-Metrics) or Clampfit (Version 10.3; Molecular device). All data are presented as the mean ± SEM. The exact sample size (e.g. the number of mice, brain slices, brains or neurons) of each experiment is indicated in the figure.

**Fig. 1:**
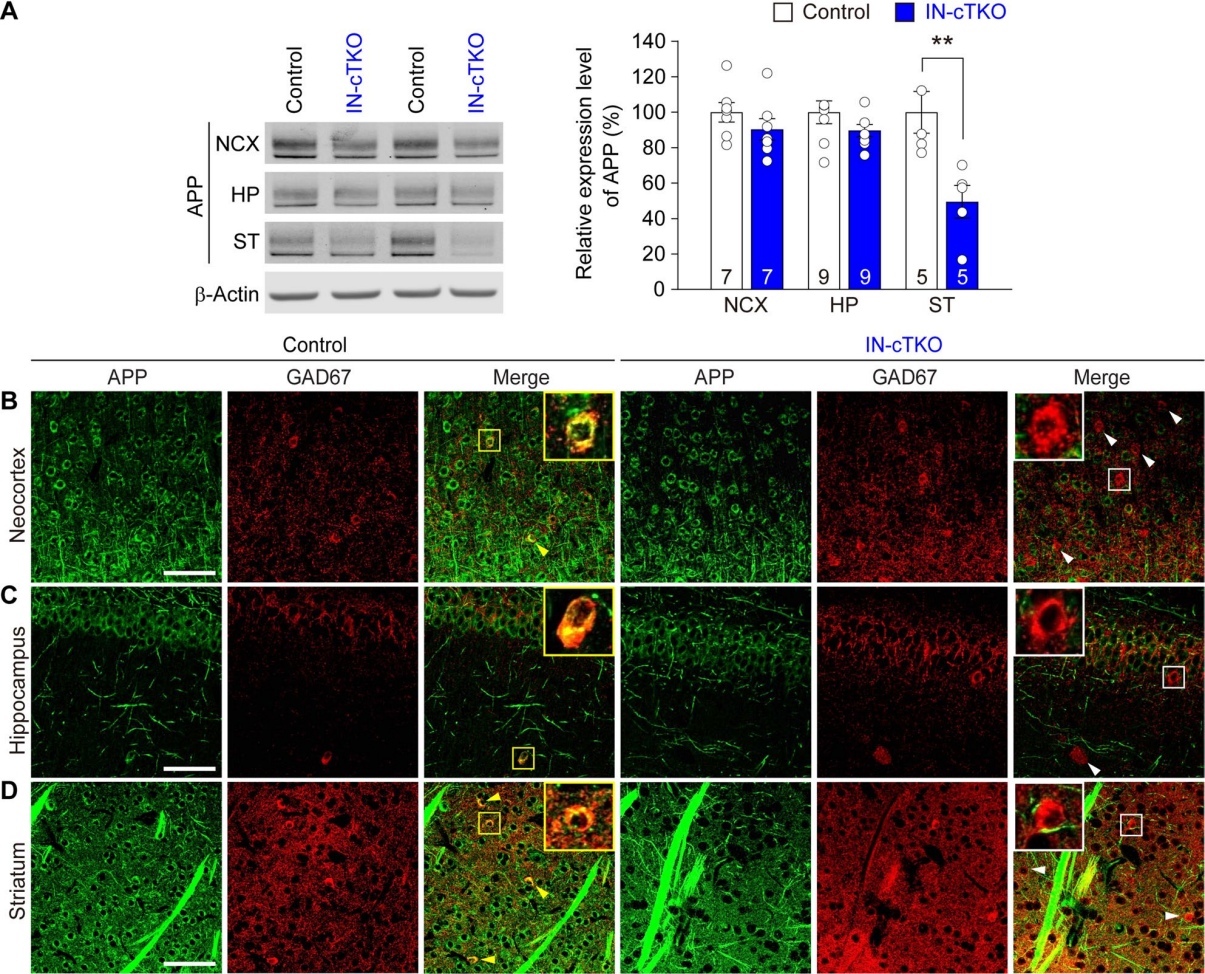
Inhibitory neuron selective inactivation of APP in the brains of IN-*APP/APLP1/APLP2* cTKO mice. (**A**) ***Left:*** Representative western blots of total protein lysates from neocortex (NCX), hippocampus (HP), and striatum (ST) of IN*-APP/APLP1/APLP2* cTKO and littermate control mice at 3 months of age. β-Actin was used as a loading control. ***Right:*** Quantification shows a significant reduction of APP in ST from IN-*APP/APLP1/APLP2* cTKO mice compared with controls (*p* = 0.009, unpaired *t*-test). APP levels in NCX (*p* = 0.262) and HP (*p* = 0.179) are not statistically different between genotypes. Control values were normalized to 100%. Numbers of mice are indicated in the bar graphs. (**B-D**) Immunohistochemistry shows abundant APP immunoreactivity (green) that co-localizes with GAD67 (red) in neocortex (**B**), stratum radiatum of hippocampal CA1 (**C**), and striatum (**D**) in control mice, whereas APP immunoreactivity is selectively eliminated from GAD67-positive interneurons in IN-*APP/APLP1/APLP2* cTKO mice (arrowheads). Insets represent higher-magnification views of the boxed regions. Scale bar: 100 μm. All data represent mean ± SEM (** *p* < 0.01)

**Fig. 2:**
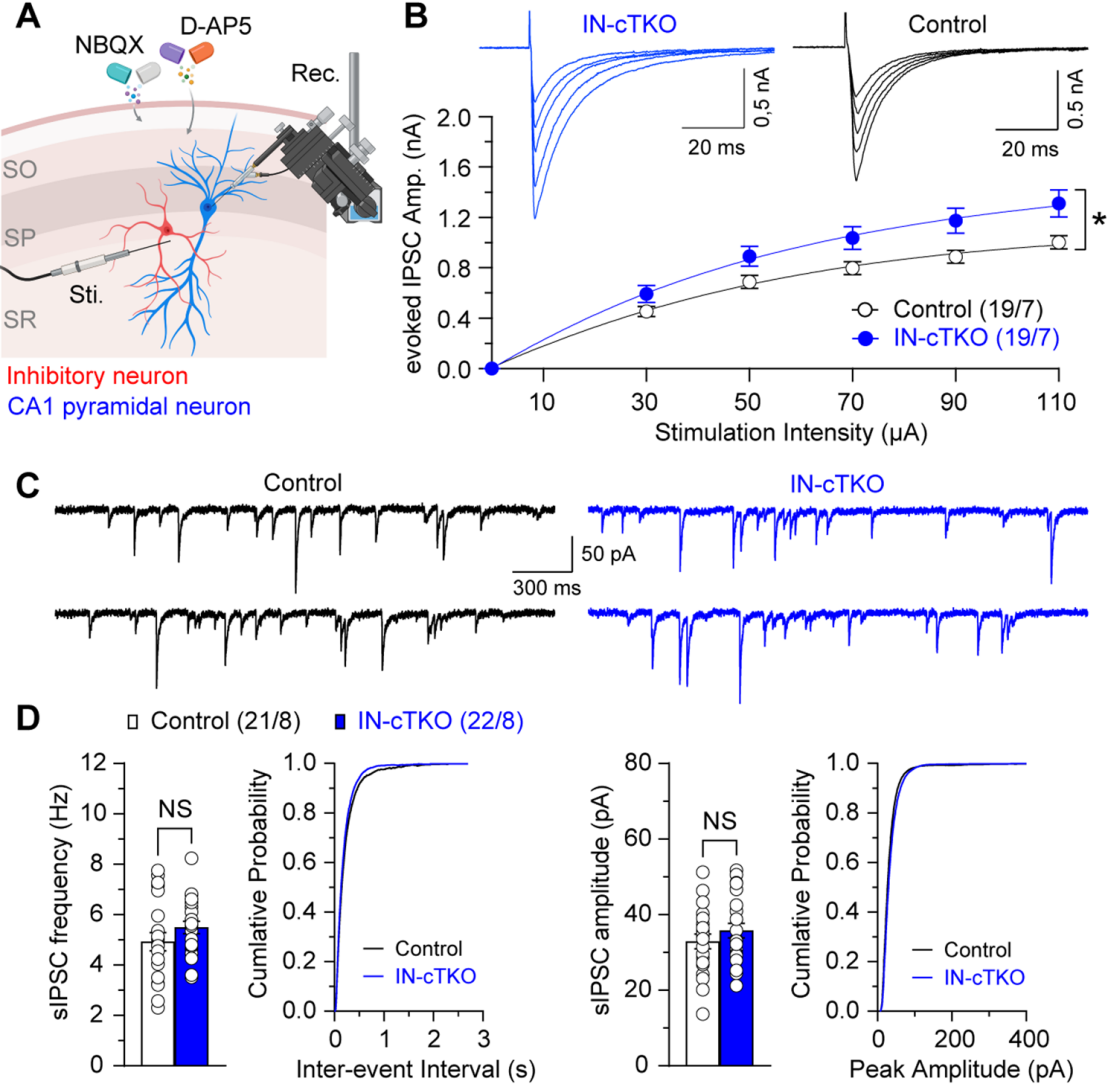
Increased GABAergic synaptic responses in hippocampal CA1 neurons of IN-*APP/APLP1/APLP2* cTKO mice. (**A**) Schematic drawing shows mono-synaptic fast GABAergic inhibition of CA1 pyramidal neurons. Local inhibitory neurons were stimulated based on their morphology and proximity to CA1 pyramidal neurons, in the presence of AMPA/NMDA receptor blockade (10 µM NBQX, 50 µM D-AP5). SO: stratum oriens, SP: stratum pyramidale, SR: stratum radiatum, Rec.: recording pipette, Sti.: stimulator. (**B**) Input/output relations of evoked GABA_A_ receptor IPSCs in slices from control and IN-*APP/APLP1/APLP2* cTKO mice. The IPSC amplitude is plotted as a function of stimulation intensity. IN-*APP/APLP1/APLP2* cTKO neurons show increase of evoked IPSC amplitudes (F_1, 36_ = 5.77, *p* = 0.02, two-way mixed-effects model). (**C**) Representative sIPSCs recorded in CA1 pyramidal neurons from control and IN-*APP* cTKO mice. (**D**) Statistical analysis indicates that IN-*APP/APLP1/APLP2* cTKO neurons exhibit unchanged sIPSC frequency and amplitude compared to controls. Cumulative inter-event interval and amplitude histograms of sIPSCs recorded in slices from control and IN-*APP/APLP1/APLP2* cTKO mice, showing comparable distributions between the two groups. All data represent mean ± SEM (* *p* < 0.05; NS: not significant). The number of neurons/mice used in each experiment is shown in parentheses

**Fig. 3:**
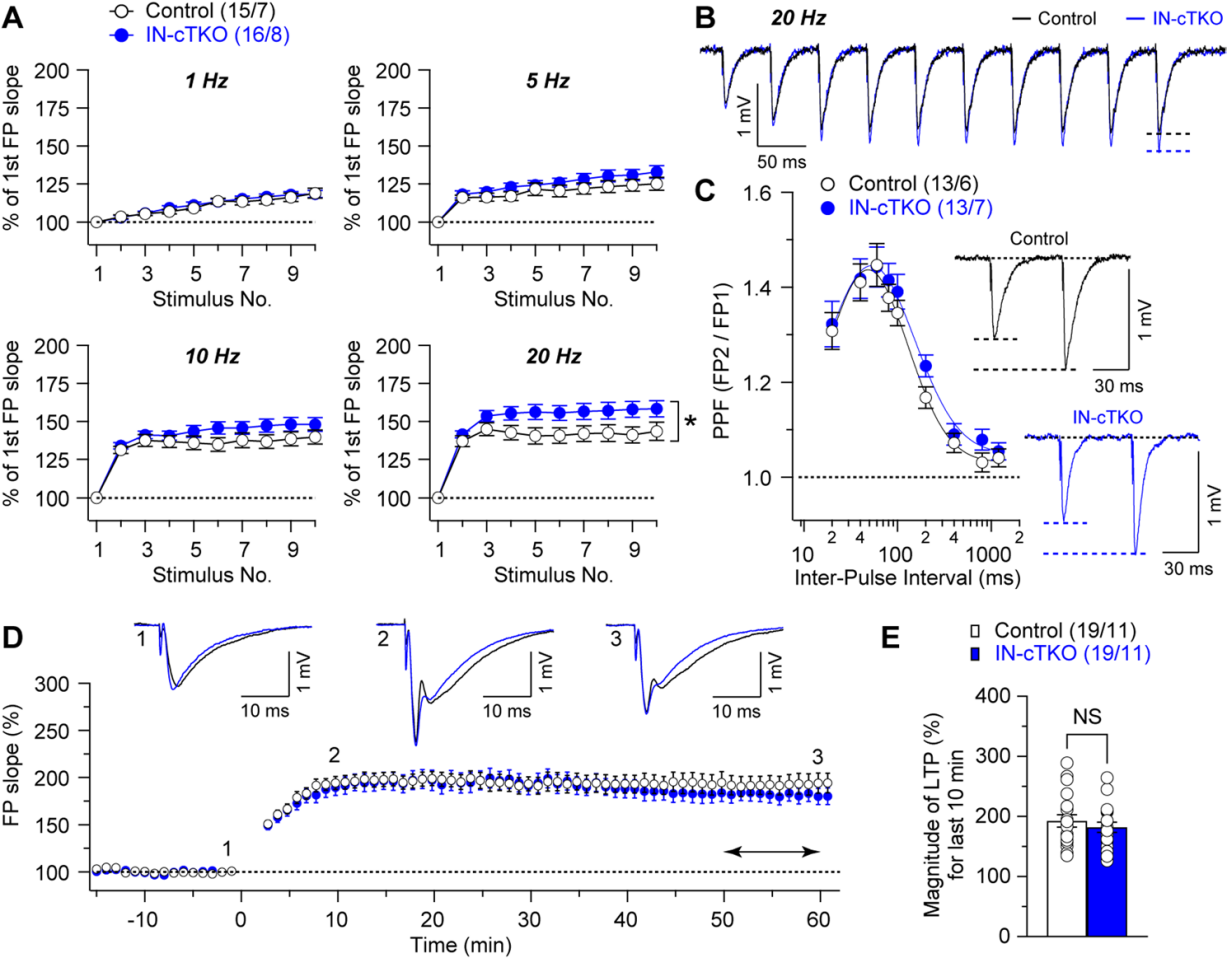
Altered short-term plasticity but normal LTP in the hippocampal Schaffer collateral pathway of IN-*APP/APLP1/APLP2* cTKO mice. (**A**) Summary graphs show that synaptic facilitation elicited by stimulus trains is enhanced, and that the enhancement is greater IN-*APP/APLP1/APLP2* cTKO mice relative to controls at 20 Hz (F_1, 29_ = 6.06, *p* = 0.02, two-way mixed-effects model). (**B**) Superimposed fEPSP traces of frequency facilitation elicited by 20 Hz stimulus train show greater enhancement in IN-*APP/APLP1/APLP2* cTKO mice relative to controls. (**C**) Averaged PPF is plotted as a function of the inter-stimulus intervals (20-1200ms), displaying normal PPF in IN-*APP/APLP1/APLP2* cTKO mice (F_1, 24_ = 0.36, *p* = 0.55, two-way mixed-effects model). Insets are representative fEPSP traces evoked by two consecutive stimuli with a 60 ms inter-pulse interval. (**D**) Normal LTP induced by TBS in IN-*APP/APLP1/APLP2* cTKO mice. Superimposed traces are averages of four consecutive responses 1 min before (1), 10 min and 60 min after (2, 3) TBS induction. (**E**) Summary graph shows the magnitude of LTP measured during the last 10 min post-induction (51–60 min) in control and IN-*APP* cTKO hippocampal slices. All data represent mean ± SEM (* *p* < 0.05, NS: not significant). The number of slices/mice used in each experiment is shown in parentheses.

**Fig. 4:**
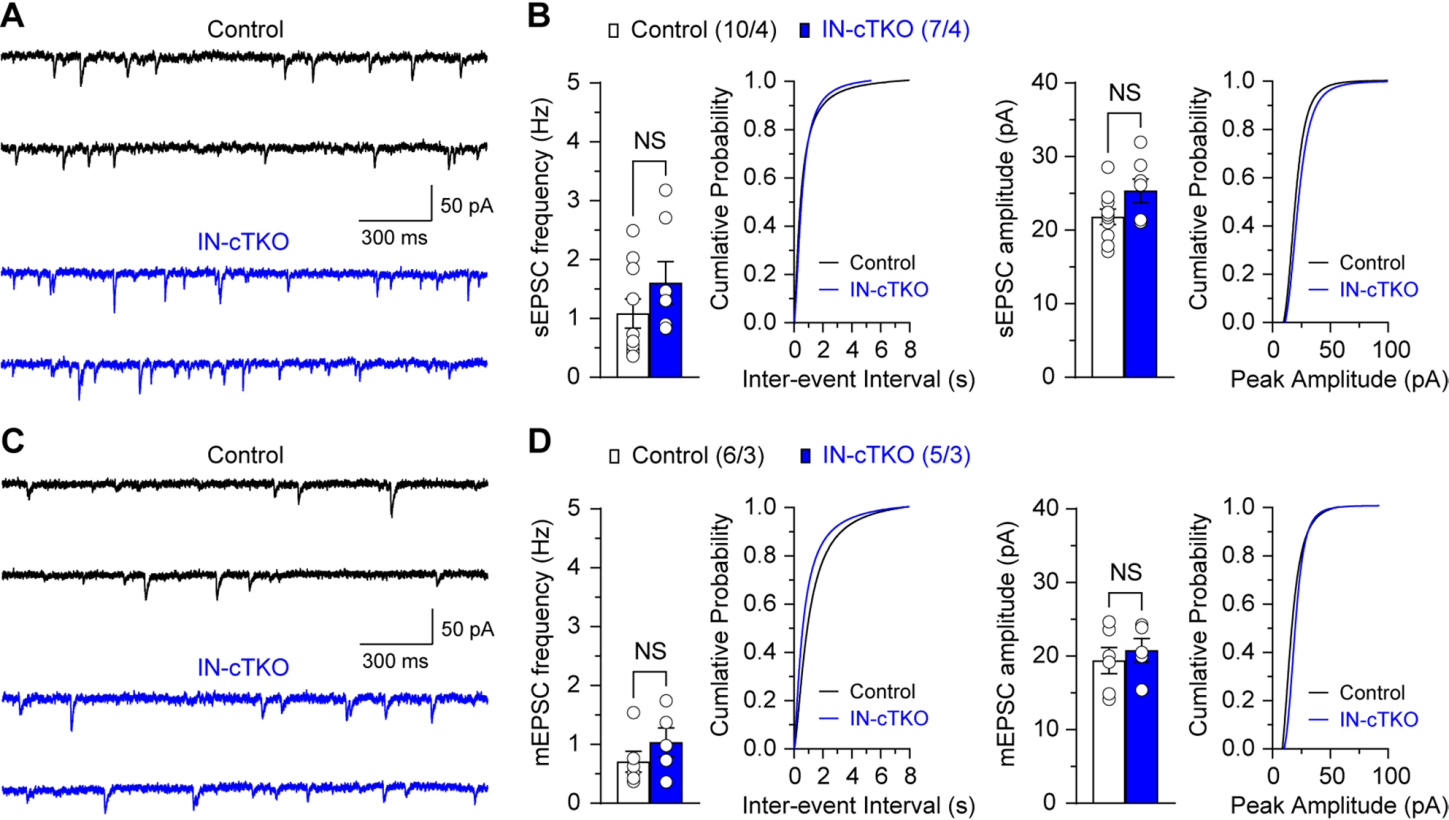
No significant genotype effect on spontaneous and miniature EPSC. (**A**) Representative sEPSCs recorded in CA1 pyramidal neurons from control and IN-*APP/APLP1/APLP2* cTKO mice at a holding potential of − 70 mV in the presence of blocker of GABA_A_ receptor (100 µM picrotoxin). (**B**) Statistical analysis did not detect a significant difference in sEPSC frequency (*p* = 0.233, unpaired *t*-test) or amplitude (*p* = 0.073, unpaired *t*-test) in IN-*APP/APLP1/APLP2* cTKO neurons. Cumulative inter-event interval and amplitude histograms of sEPSCs recorded in slices from control and IN-*APP/APLP1/APLP2* cTKO mice, showing similar distributions between the two groups. (**C**) Representative mEPSCs recorded in CA1 pyramidal neurons from control and IN-*APP/APLP1/APLP2* cTKO mice at a holding potential of − 70 mV in the presence of 100 µM picrotoxin for the blockade of GABA_A_ receptor with 1 µM TTX. (**D**) Statistical analysis indicates similar mEPSC frequency (*p* = 0.287, unpaired *t*-test) and amplitude (*p* = 0.582, unpaired *t*-test) between IN-*APP/APLP1/APLP2* cTKO and control neurons. Cumulative inter-event interval and amplitude histograms of mEPSCs recorded in slices from control and IN-*APP/APLP1/APLP2* cTKO mice, displaying comparable distributions between the two groups. All data represent mean ± SEM (NS: not significant). The number of neurons/mice used in each experiment is shown in parentheses

Statistical analyses were conducted using Student’s *t*-test for Figs. 1A, 2D, 3E and 4B, and D. For Figs. 2B and 3A, and C, statistical comparisons across genotype and stimulus conditions were performed using a two-way mixed-effects model (REML), followed by Bonferroni post hoc multiple-comparisons tests, implemented in GraphPad Prism. All statistical comparisons were performed on data from ≥ 3 biologically independent samples and replicated on different experimental days. Significance is shown as *p < 0.05, **p < 0.01 or NS (not significant).

## Results

### Generation and molecular validation of IN-*APP/APLP1/APLP2* cTKO mice

To investigate the physiological role of the APP family in inhibitory interneurons, we generated inhibitory neuron-specific *APP/APLP1/APLP2* conditional triple knockout (IN-cTKO) mice using our previously thoroughly characterized triple floxed *APP/APLP1/APLP2* mice [14] and interneuron-specific *GAD2-IRES-Cre* driver [18]. We previously demonstrated that Cre-mediated recombination results in the deletion of the floxed promoter region and/or the first exon(s) of each *APP* family gene (*APP*: 2.1 kb; *APLP1*: 2.8 kb; *APLP2*: 1.5 kb). We previously generated excitatory neuron–specific *APP/APLP1/APLP2* conditional triple knockout (EX-cTKO) mice using an excitatory neuron-specific *Camk2a-Cre* line [14], and here we employ an inhibitory neuron-specific *GAD2-IRES-Cre* line, which we previously characterized when inactivating *PS* genes selectively in inhibitory neurons [13]. We first crossed mice homozygous for the floxed *APP*, *APLP1*, and *APLP2* alleles (*fAPP/fAPP; fAPLP1/fAPLP1; fAPLP2/fAPLP2*) with *GAD2-IRES-Cre* mice to obtain *fAPP/+; fAPLP1/+; fAPLP2/+; GAD2-IRES-Cre/+* offspring. These were then bred with *fAPP/fAPP; fAPLP1/fAPLP1; fAPLP2/fAPLP2* mice to produce IN-*APP* cTKO mice (*fAPP/fAPP; fAPLP1/fAPLP1; fAPLP2/fAPLP2; GAD2-IRES-Cre/+*). IN-*APP* cTKO mice were subsequently intercrossed with floxed homozygous controls (*fAPP/fAPP; fAPLP1/fAPLP1; fAPLP2/fAPLP2*) to generate both IN-*APP/APLP1/APLP2* cTKO and control littermates for phenotypic analysis. IN-*APP/APLP1/APLP2* cTKO mice were viable and were born at the expected Mendelian ratio.

Previous studies using inhibitory neuron–specific *PS* conditional knockout (IN-*PS* cDKO) mice, generated with the same *GAD2-IRES-Cre* driver line that was used here for IN-*APP/APLP1/APLP2* cTKO mice, reported 10–30% reduction in PS1 protein levels in various brain sub-regions compared with littermate controls at 2 months of age [13]. To verify whether APP expression is similarly reduced in IN-*APP/APLP1/APLP2* cTKO mice, we performed Western blotting using protein lysates from the neocortex (NCX), hippocampus (HP), and striatum (ST) of IN-*APP/APLP1/APLP2* cTKO and control littermates at 3 months of age. APP protein levels in the cerebral cortex of IN-*APP/APLP1/APLP2* cTKO mice showed a slight, but not statistically significant, reduction compared with controls (Fig. 1A; NCX: 90.30 ± 5.68%, *p* = 0.262; HP: 89.63 ± 3.24%, *p* = 0.179; unpaired *t*-test). In contrast, APP expression in the striatum, a region enriched in GAD67-expressing interneurons, was significantly reduced by ~ 50% relative to littermate controls (Fig. 1A; *p* = 0.009; unpaired *t*-test). To verify the cell-type specificity of APP inactivation, we performed immunohistochemical analysis and observed co-localization of APP with GAD67-positive interneurons in the neocortex, hippocampus, and striatum of control mice. Notably, APP immunoreactivity was absent in GAD67-positive interneurons of IN-*APP/APLP1/APLP2* cTKO mice (Fig. 1B–D). To further confirm the cell-type specificity of APP deletion in our IN-*APP/APLP1/APLP2* cTKO mice, we performed double immunofluorescence staining for APP and the interneuron marker GAD67 across the NCX, HP, and ST (Fig. 1B–D). In control mice, APP expression was observed in both GAD67-negative neurons and GAD67-positive interneurons, as indicated by the clear co-localization in the merged images (Fig. 1B-D, insets, yellow arrowheads). In contrast IN-*APP/APLP1/APLP2* cTKO mice exhibited a prominent and selective loss of APP immunoreactivity within GAD67-positive cells (Fig. 1B–D, insets, white arrowheads). Together, these results suggest that similar to IN-*PS* cDKO mice, *GAD2-IRES-Cre* also drives inactivation of APP in interneurons of IN-*APP* cTKO mice by 3 months of age.

### Loss of APP family in inhibitory neurons increases evoked GABAergic inhibition

Although GABAergic neurons comprise only ~ 10% of hippocampal neurons, they are crucial for coordinating hippocampal network activity and plasticity [20, 21]. To investigate the physiological role of the APP family in interneurons and its impact on excitatory signaling within hippocampal networks, we assessed GABAergic inhibitory synaptic responses in IN-*APP/APLP1/APLP2* cTKO mice. First, we compared input–output (I/O) relationships for evoked GABA_A_ receptor-mediated inhibitory postsynaptic currents (IPSCs) between IN-*APP/APLP1/APLP2* cTKO mice and littermate controls. Evoked IPSCs were recorded from CA1 pyramidal neurons under voltage clamp (− 70 mV) using a high [Cl^−^] intrapipette solution with AMPA and NMDA receptors blocked (10 µM NBQX, 50 µM D-AP5). Evoked IPSCs, assayed by input-output (I/O) curves using local stimulation in stratum pyramidale, were markedly enhanced in IN-*APP/APLP1/APLP2* cTKO mice compared to controls (Fig. 2B; F_1, 36_ = 5.77, *p* = 0.02, two-way mixed-effects model). To determine whether these I/O changes reflect altered presynaptic properties and/or network activity at rest, we measured spontaneous IPSCs (sIPSCs) in CA1 neurons under the same pharmacological blockade. sIPSC frequency was unchanged (Fig. 2C and D; *p* = 0.20; unpaired *t*-test), and sIPSC amplitude also did not differ between genotypes (Fig. 2C and D; *p* = 0.30; unpaired *t*-test). Thus, APP family loss in inhibitory neurons enhances stimulus-driven inhibitory efficacy without affecting spontaneous inhibitory transmission.

### Enhanced short-train frequency facilitation in IN-*APP/APLP1/APLP2* cTKO mice

In addition to increased evoked GABAergic inhibition reflecting altered local inhibitory output onto pyramidal neurons in IN-*APP/APLP1/APLP2* cTKO mice, we examined short- and long-term synaptic plasticity by analyzing Schaffer collateral (SC) pathway–evoked excitatory field responses to further define the functional impact of APP family inactivation in inhibitory neurons. For short-term plasticity, we quantified frequency facilitation during 10-pulse trains (1–20 Hz) and paired-pulse facilitation (PPF) across inter-pulse intervals of 20–1200 ms. Frequency facilitation was more prominent in IN-*APP/APLP1/APLP2* cTKO hippocampal slices, with a significant effect at 20 Hz (Fig. 3A and B; 1 Hz: F_1, 29_ = 0.19, *p* = 0.66; 5 Hz: F_1, 29_ = 1.50, *p* = 0.23; 10 Hz: F_1, 29_ = 2.14, *p* = 0.15; 20 Hz: F_1, 29_ = 6.06, *p* = 0.02; two-way mixed-effects model). In contrast, PPF was normal in IN-*APP/APLP1/APLP2* cTKO hippocampal slices (Fig. 3C; F_1, 24_ = 0.36, *p* = 0.55, two-way mixed-effects model).

We then assessed the effects of APP family inactivation in inhibitory neurons on long-term potentiation (LTP) at SC–CA1 synapses. LTP was induced by five trains of theta-burst stimulation (TBS), quantifying changes in the initial slope of the evoked field excitatory postsynaptic potentials (fEPSPs). LTP magnitude measured 51–60 min post-induction was not significantly different between groups under our recording conditions (Fig. 3D and E; Control: 192.4 ± 10.2%, cTKO: 181.9 ± 8.5%; *p* = 0.43, unpaired *t*-test).

### No significant alteration in basal excitatory synaptic transmission in IN-*APP/APLP1/APLP2* cTKO mice

Given that enhanced frequency facilitation could reflect heightened network excitability, we next evaluated basal excitatory transmission onto CA1 pyramidal neurons under recording conditions expected to primarily reflect AMPAR-mediated synaptic currents (voltage clamp at − 70 mV; GABA_A_ receptor blocked). Under these conditions, the frequency and amplitude of spontaneous excitatory postsynaptic currents (sEPSCs) did not show a statistically significant genotype effect (Fig. 4A and B; frequency of sEPSC, *p* = 0.233, amplitude of sEPSC, *p* = 0.073; unpaired *t*-test), indicating comparable ongoing excitatory drive at rest. To further dissociate spontaneous presynaptic release from action potential (AP) – dependent activity, we recorded miniature excitatory postsynaptic currents (mEPSCs) in the presence of 1 µM tetrodotoxin (TTX). The mEPSC frequency and amplitude were also similar between groups (Fig. 4C and D; frequency of mEPSC, *p* = 0.287, amplitude of mEPSC, *p* = 0.582; unpaired *t*-test). Together, these data indicate that we did not detect a baseline difference in glutamatergic event frequency or amplitude under our recording conditions following inhibitory neuron–specific APP family deletion. In combination with the intact PPF and unchanged LTP described above, these results support the notion that the predominant phenotype reflects altered inhibitory recruitment and network dynamics rather than a baseline enhancement of excitatory synaptic transmission. However, modest genotype effects on excitatory event frequency or amplitude cannot be excluded with the current sample size, and larger cohorts would be required to rigorously detect small differences.

**Fig. 5:**
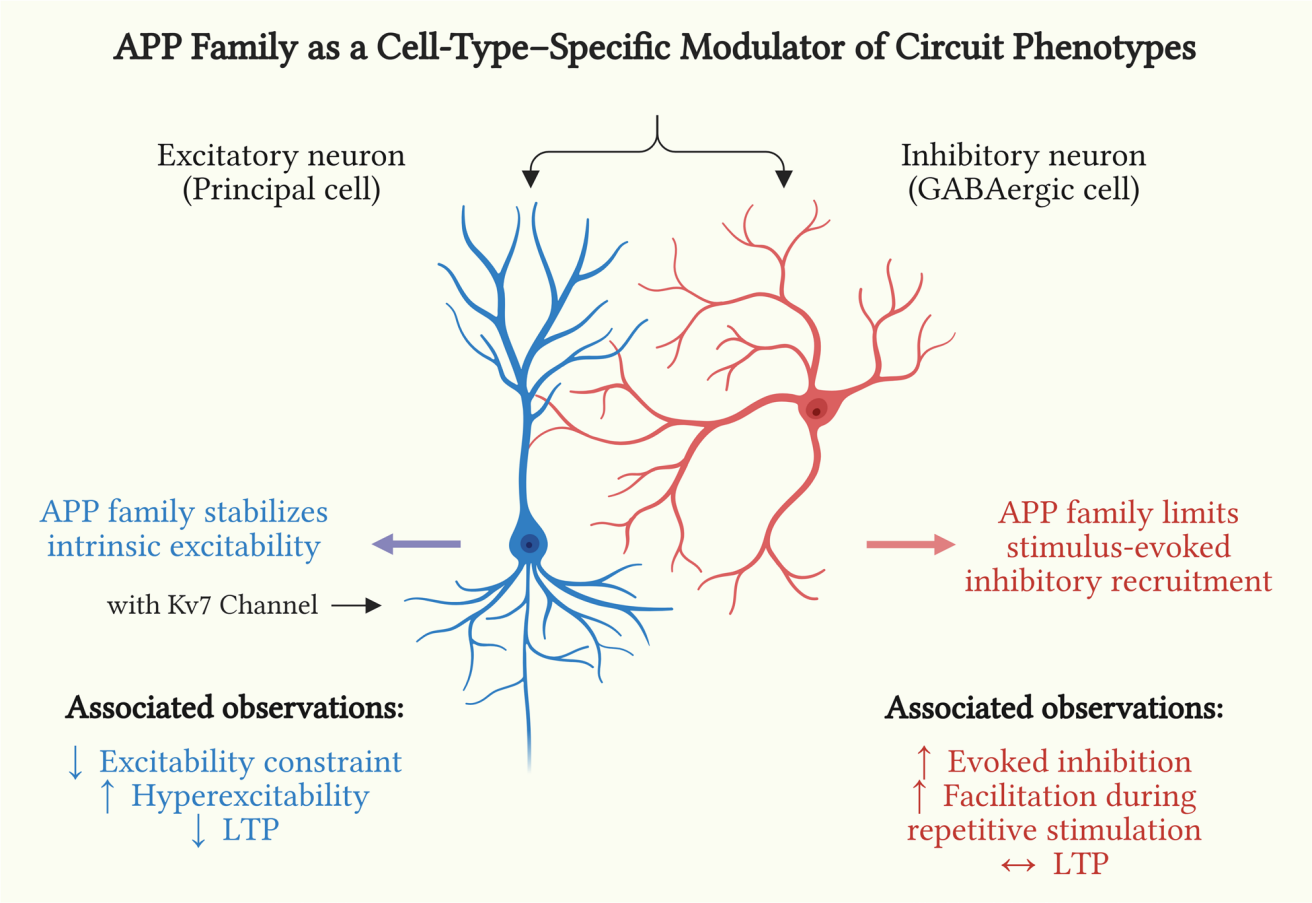
APP family as a cell-type–specific modulator of hippocampal circuit phenotypes. Schematic summarizing complementary, cell-type–specific roles of the APP family in hippocampal circuits. ***Left*** **(excitatory neuron; prior work):** APP family promote Kv7 channel function, which is thought to stabilize intrinsic excitability and supporting LTP; loss in excitatory neurons leads to hyperexcitability and impaired LTP (as shown in our previous work) [14]. ***Right*** **(inhibitory neuron; this study):** APP family may limit stimulus-evoked inhibitory recruitment; loss in inhibitory neurons is associated with increased evoked inhibition and enhances short-term facilitation during repetitive stimulation, without a statistically significant change in LTP under our conditions. This schematic is intended as a hypothesis-generating framework and does not imply that all depicted relationships have been mechanistically tested.

In excitatory-neuron *APP/APLP1/APLP2* cTKO mice, we previously observed impaired LTP, increased PPF and frequency facilitation, elevated sEPSC frequency, and intrinsic hyperexcitability of CA1 pyramidal neurons attributable to reduced Kv7 channel function [14]. Together, the two cell-type–specific manipulations suggest complementary roles: APP family in principal cells stabilizes intrinsic excitability and NMDA receptor-dependent plasticity, whereas APP family in inhibitory neurons restrains activity-dependent inhibitory recruitment and shapes short-term facilitation. Thus, we speculate that these differential contributions may be essential for preserving circuit stability and ensuring stable information flow within hippocampal circuits (Fig. 5).

## Discussion

In the current study, we demonstrate that selective deletion of APP, APLP1, and APLP2 in GABAergic neurons (IN-*APP/APLP1/APLP2* cTKO) reveals an inhibitory neuron-specific role for the APP family in regulating hippocampal inhibitory tone and short-term synaptic plasticity. IN-*APP/APLP1/APLP2* cTKO slices showed markedly enhanced amplitudes of stimulus-evoked GABA_A_ receptor-mediated IPSCs in CA1 pyramidal neurons, assayed with input–output curves (Fig. 2B), indicating stronger recruitment of inhibitory synapses during afferent stimulation. In contrast, spontaneous IPSC frequency and amplitude remained indistinguishable from controls (Fig. 2C and D), demonstrating that APP family loss in interneurons enhances stimulus-driven inhibitory efficacy without altering baseline inhibitory transmission. Basal excitatory transmission, measured by spontaneous and miniature EPSCs, did not differ significantly between genotypes (Fig. 4), although subtle effects may require larger cohorts to detect. LTP induced by theta-burst stimulation at SC–CA1 synapses was comparable between groups (Fig. 3D and E), and paired-pulse facilitation was likewise unaffected (Fig. 3C). Together, these results show that loss of APP family in inhibitory neurons enhances evoked inhibitory output and shapes short-term network dynamics while leaving long-term plasticity intact. Interestingly, we observed a specific enhancement of short-train frequency facilitation at SC–CA1 synapses in IN-*APP/APLP1/APLP2* cTKO hippocampus (Fig. 3A and B). During brief 20 Hz stimulus trains, facilitation of successive fEPSPs was significantly greater in IN-*APP/APLP1/APLP2* cTKO slices than in controls, whereas PPF was normal, suggesting that basal presynaptic release probability at excitatory synapses was unchanged. Instead, the augmented facilitation emerged selectively over the course of high-frequency trains. A plausible interpretation is that the increased feed-forward inhibition in IN-*APP/APLP1/APLP2* cTKO circuits, evidenced by the larger evoked IPSCs (Fig. 2B), suppresses the earliest responses in a train more effectively, thereby allowing subsequent responses to facilitate to a greater relative extent. In essence, stronger inhibitory recruitment early in a stimulus train reshapes the short-term excitatory dynamics recorded in CA1. Consistent with this idea, interneuron-driven changes in inhibitory timing are known to influence excitatory summation and plasticity in hippocampal circuits [12]. Accordingly, by modulating inhibitory recruitment, APP family signaling in interneurons may help set the gain and temporal filtering of excitatory neurotransmission during repetitive activity without altering the capacity for long-term synaptic modification.

These findings contrast with the phenotype observed in excitatory neuron–specific APP/APLP1/APLP2 cTKO (EX-cTKO) mice, underscoring the cell-type specificity of APP family effects. In our previous study, excitatory neuron–restricted deletion led to impaired hippocampal LTP, defective learning, and markedly increased intrinsic excitability linked to dysregulation of Kv7 (M-type) potassium channels [14]. In contrast, IN-APP/APLP1/APLP2 cTKO mice exhibited normal LTP and no overt pyramidal cell hyperexcitability, but instead showed enhanced inhibitory output. Thus, deleting APP family from excitatory versus inhibitory neurons produces complementary outcomes: the former removes a brake on excitability, leading to over-excitation and weakened potentiation, whereas the latter removes a brake on inhibitory efficacy, leading to stronger synaptic inhibition with preserved potentiation (Fig. 5). These complementary phenotypes suggest that the APP family modulates both excitatory and inhibitory aspects of hippocampal circuit activity through distinct cell-autonomous mechanisms, maintaining homeostatic equilibrium in principal cells versus interneurons [14, 22–24]. 

At the mechanistic level, the divergent effects of APP family removal in interneurons versus excitatory cells may reflect engagement of different molecular pathways. APP and its paralogs are multidomain proteins capable of both signaling and cell-adhesive functions. Notably, APLP1 has been identified as a synaptic cell adhesion molecule supporting synapse maintenance and baseline transmission [17, 25]. In interneurons, APP family could contribute to the assembly or stability of GABAergic synapses, for example by promoting proper alignment of presynaptic terminals with postsynaptic receptors or by participating in trans-synaptic complexes that regulate release probability. Loss of such adhesion/signaling molecules from interneurons might therefore alter the efficacy with which interneurons are recruited by network activity. The increased evoked IPSCs in IN-*APP/APLP1/APLP2* cTKO mice are consistent with a scenario in which inhibitory synapses become more readily engaged when APP family is absent, yet this occurs without detectable change in baseline inhibitory activity (sIPSCs) (Fig. 2C and D), implying that network compensation maintains resting inhibition while differences emerge during active recruitment of synapses. One possibility is that APP family–dependent adhesion or signaling organizes presynaptic release sites or influences short-term presynaptic calcium dynamics at inhibitory terminals, and that in its absence, interneurons exhibit facilitated release or enhanced synchronization during evoked responses. Although the precise molecular mechanism remains to be determined, our data highlights that APP family-dependent adhesion/signaling in inhibitory neurons fine-tunes inhibitory strength in an activity-dependent manner, preventing an overshoot of inhibition during bursts of network activity.

It is informative to compare the present results with our previous findings from IN-*PS* cDKO mice, which were generated with the same *GAD2-IRES-Cre* driver used here [12, 13]. IN-*PS* cDKO mice exhibited the opposite synaptic phenotype: reduced GABA release efficacy and consequent enhancement of LTP at SC–CA1 synapses [12], accompanied by age-dependent interneuron loss and gliosis [13]. In IN-*APP/APLP1/APLP2* cTKO mice, by contrast, we observed strengthened evoked inhibition and no change in LTP, with no evidence of interneuron loss at the ages studied (data not shown). This dichotomy suggests that APP family proteins and PS influence inhibitory neuron function through distinct pathways. PS, as part of the γ-secretase complex, is required cell-autonomously for interneuron survival and for maintaining baseline GABAergic transmission, likely through processing of substrates essential for interneuron health. APP family proteins, themselves substrates of γ-secretase, appear to modulate the synaptic output of interneurons without affecting their viability. The fact that eliminating APP family increases inhibitory strength, while eliminating PS decreases it, reinforces the concept that multiple parallel molecular systems converge to regulate inhibitory efficacy and thereby govern excitation–inhibition (E/I) balance in hippocampal circuits.

Our findings also have implications for understanding E/I imbalances in AD. Aberrant cortical and hippocampal activity, disrupted oscillations, and epileptiform events emerge early in AD and have been linked to impaired inhibitory control [4, 6, 11]. Interventions that enhance interneuron function can normalize network rhythms and improve behavioral outcomes [3, 5, 10, 26], supporting a causal role for inhibitory control in disease-relevant circuit dysfunction. In this context, APP is typically considered primarily as the precursor to Aβ; however, our data highlight that full-length APP family proteins also participate in the physiological regulation of inhibitory output. Notably, inhibitory neuron–specific APP family inactivation increased evoked inhibition, which differs in direction from the reduced inhibition often implicated in AD, suggesting that AD-associated inhibitory deficits likely arise from additional disease processes such as neurodegeneration, Aβ/tau toxicity, and neuroinflammation. Nevertheless, defining the normal, cell-type–specific roles of APP family signaling provides a framework for interpreting how alterations in APP processing or related pathways could reshape circuit stability during disease progression.

Several limitations of the present study merit consideration. First, our recordings were obtained from CA1 pyramidal neurons and therefore do not resolve which inhibitory neuron subtypes are engaged, whether interneuron intrinsic excitability is altered, or whether quantal properties of GABA release are changed. Future studies using mIPSC recordings, whole-cell recordings from genetically identified interneurons, and subtype-specific targeting will be required to determine whether the enhanced evoked inhibition reflects altered synapse number, differential recruitment of specific inhibitory neuron populations, and/or changes in presynaptic release dynamics during repetitive activity. Second, because short-term plasticity and LTP were assessed under intact inhibition, complementary experiments performed under GABA_A_ receptor blockade would help separate inhibitory-network contributions from excitatory synaptic mechanisms. Third, prior work has implicated APP proteins in excitatory synapse function and AMPA receptor trafficking [15, 16, 27, 28], raising the possibility that changes in excitatory drive onto inhibitory neurons could contribute to the phenotypes observed here. In our excitatory neuron-specific cTKO study, AMPA receptor-mediated SC–CA1 input–output relationships were normal at 3 months, and GABA_A_ receptor–mediated IPSCs in CA1 pyramidal neurons were unchanged [14], indicating that APP family loss can produce selective physiological phenotypes without generalized disruption of baseline transmission at this age. Nevertheless, subtle and/or age-dependent alterations in excitatory drive onto inhibitory neurons cannot be excluded, and Cre-dependent labeling strategies could enable direct recordings from targeted interneurons to evaluate this possibility. Finally, loss of APLP1 and APLP2 was not directly quantified at the cell-type level but is genetically inferred from the presence of floxed alleles and *GAD2-IRES-Cre*-driven recombination [13, 14].

## Data Availability

The datasets generated and/or analyzed during the current study are included in the manuscript.

## Abbreviations

ACSF: Artificial cerebrospinal fluid
AD: Alzheimer’s disease
AMPA: α-amino-3-hydroxy-5-methyl-4-isoxazolepropionic acid
AP: Action potential
APLP1: Amyloid precursor-like protein 1
APLP2: Amyloid precursor-like protein 2
APP: Amyloid precursor protein
CA1: Cornu Ammonis 1 area of hippocampus
CA3: Cornu Ammonis 3 area of hippocampus
Cl^−^: Chloride ion
EPSC: Excitatory postsynaptic current
fEPSP: Field excitatory postsynaptic potential
GABA: Gamma-aminobutyric acid
GABA_A_ receptor: Gamma-aminobutyric acid receptor subtype A
GAD2: Glutamate decarboxylase2
IN-*APP/APLP1/APLP2* cTKO: Inhibitory neuron-specific *APP/APLP1/APLP2* conditional triple knockout
IN-*PS* cDKO: Inhibitory neuron-specific* Presenilin* conditional double knockout
I/O: Input/output
IPSC: Inhibitory postsynaptic current
LTP: Long-term potentiation
NMDA: N-methyl-D-aspartate
PPF: Paired-pulse facilitation
SC: Schaffer collateral
sIPSCs: Spontaneous inhibitory postsynaptic currents
TBS: Theta burst stimulation
